# Ultrasound for diagnosis of postpartum retained products of conception—How accurate we are?

**DOI:** 10.1186/s12884-023-05863-4

**Published:** 2023-08-10

**Authors:** Yael Yagur, Liron Jurman, Omer Weitzner, Nissim Arbib, Ofer Markovitch, Zvi Klein, Yair Daykan, Ron Schonman

**Affiliations:** 1 Department of Obstetrics and Gynecology, Meir Medical Center, Kfar Saba, Israel; 2 Faculty of Medicine, Tel Aviv University, Tel Aviv, Israel

**Keywords:** Hysteroscopy, Positive predictive value, PPV, Retained products of conception, RPOC

## Abstract

**Objective:**

Postpartum retained products of conception (RPOC) can cause short- and long-term complications. Diagnosis is based on ultrasound examination and treated with hysteroscopy. This study evaluated the size of RPOC that can be related to a positive pathology result for residua.

**Materials and methods:**

This retrospective cohort study included women who underwent hysteroscopy for postpartum RPOC diagnosed by ultrasound, 4/2014–4/2022. Demographics, intrapartum, sonographic, intraoperative, and post-operative data were retrieved. We generated a ROC curve and found 7 mm was the statistically sonographic value for positive pathology for RPOC. Data between women with sonographic RPOC ≤ 7 mm and > 7 mm were compared. Positive and negative predictive values were calculated for RPOC pathology proved which was measured by ultrasound.

**Results:**

Among 212 patients who underwent hysteroscopy due to suspected RPOC on ultrasound, 20 (9.4%) women had residua ≤ 7 mm and 192 (90.6%) had residua > 7 mm. The most common complaint was vaginal bleeding in 128 cases (60.4%); more so in the residua > 7 mm group (62.5% vs. 40%, p = .05). Among women with residua ≤ 7 mm, the interval from delivery to hysteroscopy was longer (117.4 ± 74.7 days vs. 78.8 ± 68.8 days, respectively; p = .02). Positive pathology was more frequent when residua was > 7 mm. PPV for diagnosis of 7 mm RPOC during pathology examination was 75.3% and NPV 50%.

**Conclusions:**

Sonographic evaluation after RPOC showed that residua > 7 mm was statistically correlated with positive RPOC in pathology and PPV of 75% and NPV of 50%. Due to the high NPV and low complication rate of office hysteroscopy, clinicians should consider intervention when any RPOC are measured during sonographic examination to reduce known long-term complications.

## Introduction

Retained products of conception (RPOC) are defined as trophoblastic tissue in the uterine cavity. The incidence ranges from 3 to 6% in postpartum studies [1, 2]. The clinical characteristics of RPOC include prolonged vaginal bleeding and lower abdominal pain. However, it can be asymptomatic and discovered throughout routine ultrasound exploration [3, 4].

Sonographic diagnosis is usually based on endometrial thickness or echogenic mass, with positive Doppler examination. Endometrial fluid is part of the sonographic diagnosis [5–8]. Studies report various cut-off values for endometrial thickness and size of echogenic mass for predicting RPOC by ultrasound and the need for intervention [8, 9]. Highly suspicious ultrasound scans confirmed the diagnosis in 71% [9]. Endometrial thickness cut-off value of 10 mm has a reported sensitivity of > 80% for RPOC [10].

Treatment for RPOC is usually hysteroscopy. Hysteroscopy appears to have low complication and adhesion rates and is related to high rates of subsequent pregnancies [11, 12]. Suction curettage can be used depending on the amount of vaginal bleeding and the size of the placental remnant [11, 13]. One study reported a 91% success rate with operative hysteroscopy for postpartum RPOC, with 7.5% intrauterine adhesions and 83% fertility rate [3].

Untreated postpartum RPOC can lead to immediate and long-term complications and sequelae. Immediate outcomes can be infection and massive vaginal bleeding. Long-term outcomes can include intrauterine adhesions, further abnormal placentation and infertility; therefore, prompt diagnosis and treatment are essential [14–16].

The primary purpose of this study was to evaluate the size of RPOC diagnosed in a detailed ultrasound that can be related to a positive pathology result for residua following hysteroscopy.

## Methods

### Patients

This retrospective, monocenter cohort study included women who were admitted to the Obstetrics and Gynecology Department at Meir Medical Center for an elective surgical hysteroscopy due to suspected sonographic RPOC following delivery, during 2014–2021.

The study included women with an ultrasound-based suspected diagnosis of RPOC. Examinations were performed by a certified sonographer. The indication for an ultrasound following delivery was based on a wide range of conditions, including prolonged postpartum vaginal bleeding or abdominal pain. Some were diagnosed during routine follow-up following manual lysis of placenta during labor or revision of uterine cavity based on clinical suspicion of RPOC after the third stage of labor. Others were diagnosed during routine postpartum follow-up 4 to 8 weeks after delivery. Women with acute endometritis or emergent procedures were excluded from the study.

### Surgical hysteroscopy procedure

Patients who were 4–8 weeks postpartum were admitted for surgical hysteroscopy the morning of procedure. Based on the clinical presentation, an ultrasound was performed to verify the diagnosis and accurately characterize the findings. Sonograms included information on the location and size of the residua and Doppler findings. Surgical hysteroscopy was performed for all suspected sonographic RPOC regardless of the size of the residua or echogenic mass, with no specific cut-off values. The procedure performed by one a senior physician with more than one-year of experience performing surgical hysteroscopies. The RPOC were resected using a bipolar electrical loop using only blunt resection without electricity [17].

Intrauterine contents were sent for pathologic examination. Tissue characterized by pathologists as products of conception was defined as a positive pathology result.

### Data

Data collected from electronic medical records included demographics, delivery mode, delivery complications (postpartum hemorrhage [PPH], uterine revision or manual removal of placenta), symptoms of prolonged vaginal bleeding, interval from delivery to hysteroscopy, pathology of uterine contents and complications during the procedure.

Sonographic size of the residua was determined based on the largest diameter of 3 dimensions. To find the value for positive pathology for RPOC, we generated a ROC curve, which indicated the sonographic value for positive pathology of suspected RPOC. This indicates that there is a statistically significant difference between patients with ultrasound RPOC less or above the sonographic value found for discovering RPOC on pathology evaluation. Women’s characteristics, delivery information, clinical and ultrasonographic data and outcomes were compared between patients with residua of founded value.

To further understand the result in our population for the sonographic diagnostic result, positive predictive value (PPV) and negative predictive value (NPV) were calculated for each measured size of residua on ultrasound scan.

This study was performed in accordance with the Principles of the Declaration of Helsinki. It was approved by the Meir Medical Center Human Investigation Ethics Committee (number MMC-087-16). Due to the retrospective nature of the study, informed consent was not required.

### Statistical analysis

Patients’ characteristics were compared between the control and the study groups, using student t-test for continuous variables, and chi-square or Fisher’s Exact Test for categorical variables. p-values ≤ 0.05 were considered significant. Data are presented as numbers and percentages for categorical variables, and as means and standard deviations for continuous variables. All statistical analyses were performed using SPSS Statistics for Windows (IBM Corp., Armonk, NY).

## Results

During the study period, 212 women who were admitted for an elective surgical hysteroscopy met the inclusion criteria. Residua size ranged from 4 to 78 mm. Mean residua size was 21.3 ± 15.4 mm and median 19 mm. Average age was 33.0 ± 4.9 years, BMI (kg/m^2^) 23.7 ± 4.6, 96 (45.3%) were primigravidae, 12 (5.6%) patients had a history of RPOC and 157 (74.1%) had a vaginal delivery. During hysteroscopy, the operating surgeon clinically diagnosed RPOC in 192 (90.6%) cases. RPOC from the surgical hysteroscopy was confirmed by pathology in 155 (73.1%) cases.

A ROC curve which indicated the sonographic value for positive pathology of suspected RPOC was 7 mm. For study purposes, data was compared between patients with RPOC ≤ 7 mm and > 7 mm.

Twenty (9.4%) women were in the group of residua ≤ 7 mm and 192 (90.6%) in the group of residua > 7 mm. Baseline demographics (Table 1) did not differ significantly between groups in terms of age, smoking status, BMI (kg/m^2^), parity, history of RPOC, mode of delivery gestational age at delivery and manual uterine lysis following delivery. Previous cesarean delivery was less frequent in the group with larger residua (p = .03). Uterine revisions occurred more often in the group with smaller RPOC ( p = .005).

**Table 1:** Demographic characteristics of the cohort

Characteristic	RPOC ≤ 7 (n = 20)	RPOC > 7 (n = 192)	P-value
Age (years)	33.7 ± 6.1	33.0 ± 4.8	0.55
Smoker	2 (18.2%)	16 (12.6%)	0.64
Body mass index (kg/m^2)^	23.7 ± 3.6	23.7 ± 4.70	0.85
Primigravida	9 (45%)	89 (45.3%)	
History of RPOC	3 (15%)	9 (4.7%)	0.09
Gestational age at delivery	38 + 3 ± 2.5	38 + 6 ± 2.2	0.63
Mode of Delivery			
Normal vaginal delivery	14 (70%)	143 (74.9%)	0.64
Vacuum extraction	2 (15.8%)	16 (8.4%)	0.39
Cesarean delivery	2 (10%)	33 (17.3%)	0.54
Number of previous cesarean sections			
No history of cesarean section	20 (100%)	156 (81.3%)	0.03
One or more cesarean sections	0 (0%)	36 (18.8%)	
Manual lysis	2 (10%)	28 (14.7%)	0.75
Uterine revision	7 (35%)	23 (12%)	0.005
Postpartum hemorrhage	5 (25%)	29 (15.2%)	0.26

Data are shown as number (%), mean ± standard deviation or median (range), as appropriate. RPOC, retained products of conception

Table 2 presents clinical and ultrasonographic characteristics of the study groups. Compared to women in the ≤ 7 mm group, patients in the > 7 mm group had increased rates of prolonged postpartum vaginal bleeding (40% vs. 62%, respectively; p = .05), longer interval from delivery to hysteroscopy (117.4 ± 74.7 days vs. 78.8 ± 68.8 days, respectively; p = .02) and more had positive pathology results (75.5% vs. 50% respectively; p = .01). No significant differences were found in uterine wall location of RPOC, Doppler during ultrasound scan before hysteroscopy or complications during the procedure. Even though the > 7 mm group experienced more cases of massive bleeding (0.5% vs. 0%, respectively; p = .61) and uterine perforation (4.2% vs. 0% respectively; p = .61), the differences were not statistically significant.

**Table 2:** RPOC clinical and ultrasonographic characteristics

Variable	RPOC ≤ 7 mm (n = 20)	RPOC > 7 mm (n = 192)	p-value
Prolonged vaginal bleeding postpartum, before hysteroscopy	8 (40%)	120 (62.5%)	0.05
Abdominal pain	2 (10%)	18 (9.4%)	1
Interval from delivery to hysteroscopy, days	117.4 ± 74.7	78.7 ± 68.8	0.02
Location of RPOC			0.67
Anterior wall	3 (37.5%)	37 (25.9%)	
Posterior wall	3 (37.5%)	54 (37.8%)	
Right wall	0 (0%)	10 (7%)	
Left wall	1 (12.5%)	12 (8.4%)	
Fundus	1 (12.5%)	28 (19.6%)	
Doppler	4 (45.5%)	104 (63.4%)	0.23
Positive pathology	10 (50%)	145 (75.5%)	0.01
Difficulty during separation	2 (11.1%)	14 (7.3%)	0.63
Complications during procedure			
Massive bleeding	0 (0%)	1 (0.5%)	0.61
Uterine perforation	0 (0%)	8 (4.2%)	0.61

Data are shown as number (%), mean ± standard deviation or median (range), as appropriate. RPOC, retained products of conception

**Fig. 1:**
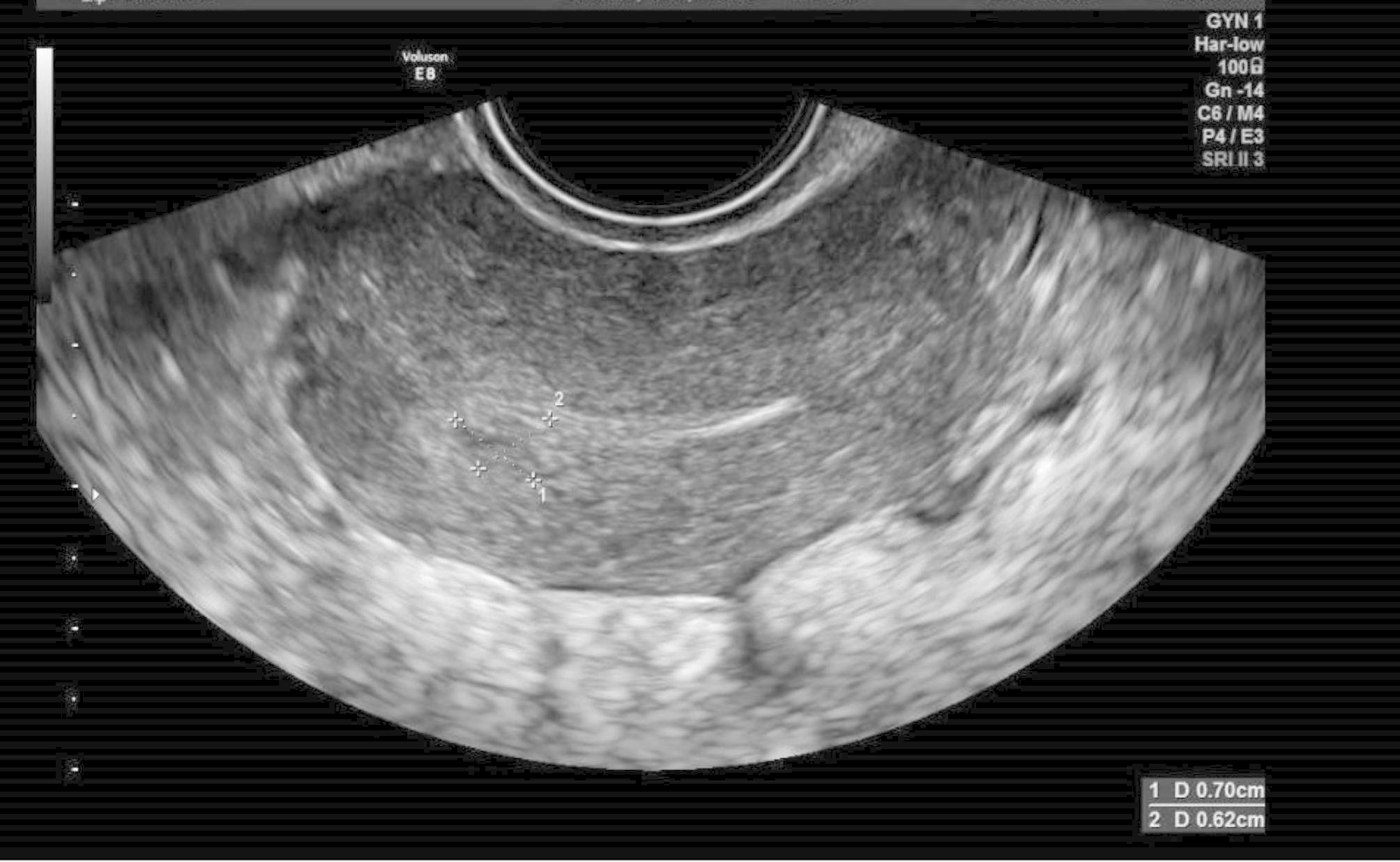
Sonographic scan for RPOC with positive pathology result. An ultrasound scan which reveals a 7 mm postpartum RPOC in an asymptomatic patient, with a positive pathological result confirming the presence of intrauterine residual remnant

Figure 1 reveals an ultrasound scan with a 7 mm postpartum RPOC in an asymptomatic patient, with a positive pathological result confirming the presence of intrauterine residual remnant.

We evaluated the PPV and NPV for the maximum size of each dimension measured on ultrasound scan for predicting positive pathology results for RPOC (Table 3). For example, the PPV for diagnosing RPOC by pathology for > 7 mm found on ultrasound examination was 75.3%, while the NPV for diagnosing RPOC by pathology for ≤ 7 mm, was 50%. Table 3 demonstrates the values for each sonographic RPOC size. The PPV for pathological confirmation was higher when the size demonstrated by the ultrasound was RPOC ≥ 42 mm was 100%. Table 3 demonstrates that even with small RPOC identified by ultrasound, the NPV was usually 30.8-50%.

**Table 3:** PPV and NPV for positive pathology results for sonographic suspected RPOC following delivery

Variable	Possitive Predictive Value	Negative Predictive Value
Diameter of RPOC (mm)		
4	73.1%	30.8%
6	74.2%	43%
7	75.3%	50%
8	78.3%	60%
9	78.7%	55.6%
10	80.2%	52.1%
11	81.8%	52.8%
12	82.8%	52.5%
13	82.8%	47.8%
14	83.3%	45.9%
15	84.7%	45.7%
16	86.1%	44.9%
17	85.9%	41.8%
18	85.6%	40.6%
19	87.1%	39.6%
20	87.1%	37.8%
21	87.1%	36.2%
22	86.1%	35.1%
23	88.2%	35.8%
24	90%	35%
25	93%	35%
27	94.8%	35%
28	94.6%	33.7%
29	93.8%	33.1%
30	93.6%	32.7%
31	97.4%	32.4%
32	97.4%	32.1%
33	97.3%	31.8%
34	96.8%	30.1%
35	100%	27.3%
36	96.3%	30.2%
37	95.5%	29.5%
38	95%	29.2%
39	93.8%	28.6%
40	93.3%	28.4%
41	92.9%	28.3%
42	100%	28.5%
44	100%	28.3%
45	100%	28.1%
46	100%	27.9%
47	100%	27.8%
53	100%	27.6%
60	100%	27.4%
78	100%	27.1%

**Fig. 2:**
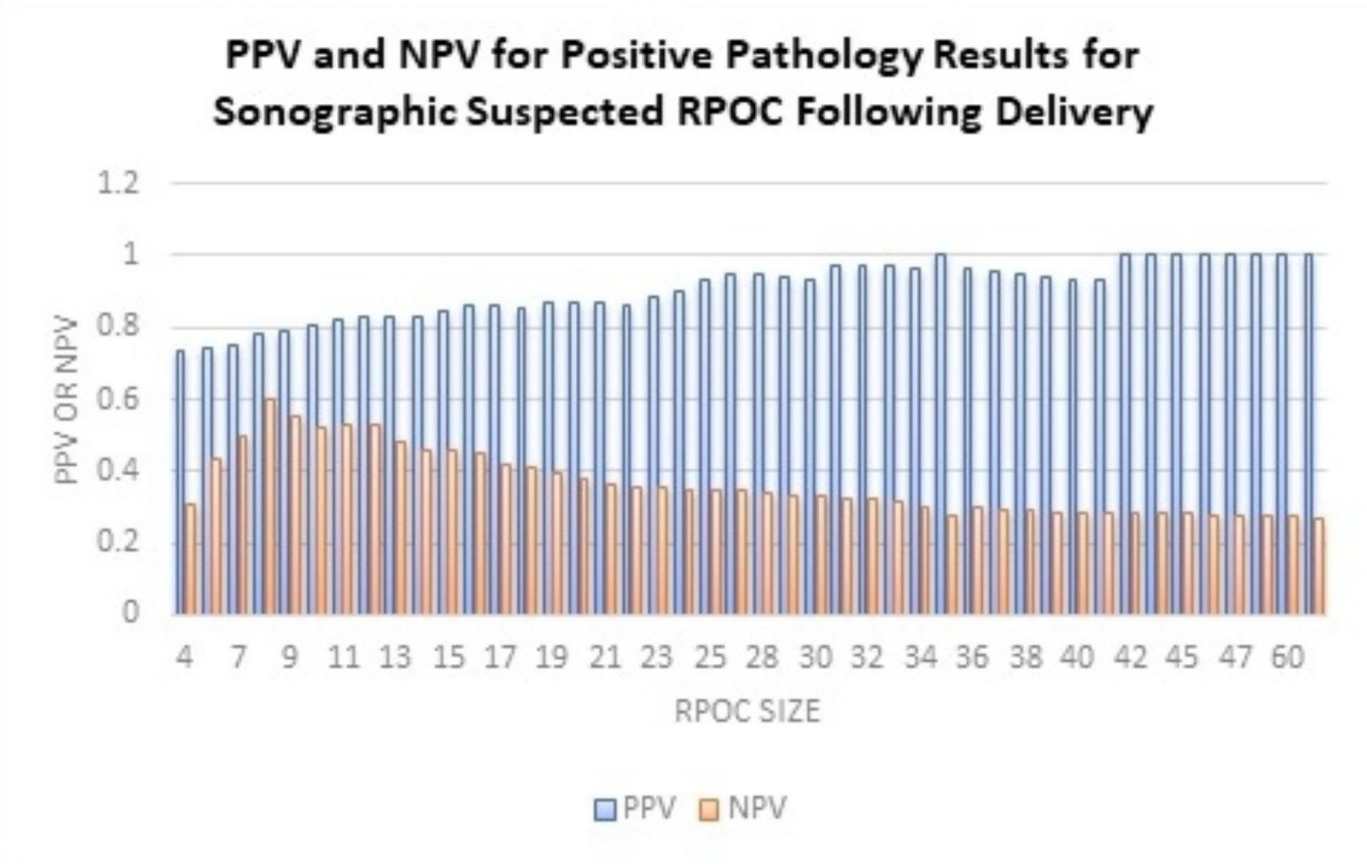
PPV and NPV for Positive Pathology Results for Sonographic Suspected RPOC Following Delivery. This figure graphically illustrates the positive predictive value (PPV) and negative predictive value (NPV) of a positive pathology result for suspected sonographic retained products of conception (RPOC) following delivery. PPV for all residual sizes is higher than 73% and NPV values show similarities across most cases

Figure 2 graphically illustrates the positive predictive value (PPV) and negative predictive value (NPV) of a positive pathology result for suspected sonographic retained products of conception (RPOC) following delivery. PPV for all residual sizes is higher than 73% and NPV values show similarities across most cases, supporting the accuracy of the sonographic results. This strengthens the permissive approach of hysteroscopy for all sizes of RPOC detected more than 8 weeks after delivery.

**Fig. 3:**
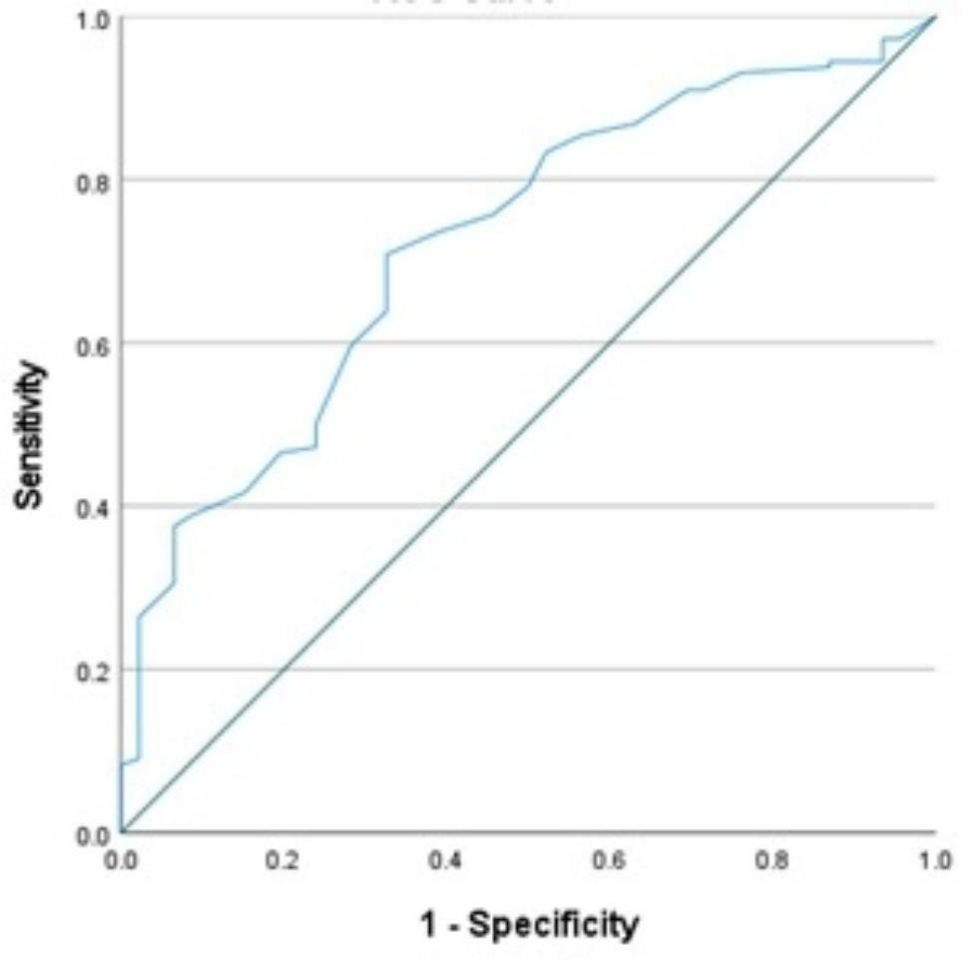
Receiver operating curves (ROCs) for positive pathology result based on sonographic RPOC. Receiver operating curves (ROCs) of positive pathology result for sonographic examination showed residual content 4–8 weeks after delivery with area under the curve (AUC) of 72.4%, (95% CI 64–84%, p < .001)

Figure 3 displays the area under the curve (AUC) for the sonographically examined values of RPOC, which predict positive pathology. The AUC was found to be significant at 72.4% (95% CI 64–84%, p < .001).

## Discussion

The purposes of this study was to evaluate the size of RPOC diagnosed by advanced ultrasound techniques and its correlation with a positive pathology result for residua and to evaluate the PPV and NPV for each RPOC sonographic value. This study showed that residua > 7 mm diagnosed on ultrasound scan are associated with positive pathology results following hysteroscopy in postpartum patients, with a PPV of 75% and NPV of 50%. In addition, longer duration from delivery to hysteroscopy was correlated with smaller RPOC and shorter duration of vaginal bleeding postpartum.

The predictive value of proven diagnosis for suspected sonographic RPOC during hysteroscopy following delivery is not well-established. One study showed that the diagnosis of RPOC should be based on the presence of an echogenic mass on sonographic scan, with positive Doppler flow. They had an 82% confirmation rate for RPOC during hysteroscopy for patients considered at-risk [8]. Our study showed that 73.1% of patients with suspected sonographic RPOC had a positive pathology report. Studies also showed that doppler was reported to be important for improving diagnostic accuracy [5, 18]. It is worth mentioning that 63.4% of patients in the group of RPOC > 7 mm had a positive Doppler flow and 51% of the entire cohort. Other studies evaluating clinical parameters for diagnosis reported that the combination of endometrial hyperechogenic mass and clinical parameters such as pain, and bleeding were not predictive of RPOC. Maternal age and vaginal delivery were significantly correlated with RPOC postpartum [19].

Based on the literature, we can understand that no specific size of RPOC can be predictive. Endometrial thickness > 10 mm can be diagnostic but is less accurate than an echogenic mass (7% sensitivity vs. 60–80%, respectively) [10]. Our findings are consistent with previous information on of the importance of an echogenic mass, while adding a new perspective on the concept of the size. An echogenic mass with a sonographic RPOC > 7 mm is highly suspicious for RPOC and was confirmed on histologic exam with a PPV of 75%. This finding encourages us to freely refer women who meet these criteria under the premise that there is a high correlation between sonographic evaluation and histologic findings. In contrast, the 50% NPV for cases with residua ≤ 7 mm indicates that under these conditions, a substantial number of patients will still have positive histology. Our data suggest that even with a very small sonographic echoic mass, the chance for true RPOC exists and a hysteroscopy should be considered.

Hysteroscopy is an office procedure with a low complication rate, particularly for small intrauterine findings of < 5 mm. There is no clear information in the literature regarding the influence of small, asymptomatic RPOC or the benefit of removing residua of any size. However, our findings support the practice of office hysteroscopy even in cases of small RPOC. Due to very low complication rates from office procedures, it is reasonable to be permissive in the use of hysteroscopy for any size residua [20].

In the current study, all patients were admitted 4–8 weeks after delivery. Our departmental policy is to wait this period for two reasons. First, for safer procedures. In addition, we believe that many cases of RPOC resolve spontaneously. When we see even a small echoic mass, hysteroscopy should be the next step in treatment.

In this study, we also evaluated risk-factors that can be related to the presence of RPOC. Patients who previously underwent uterine cavity exploration immediately postpartum due to suspected RPOC, were more likely to present with sonographic RPOC ≤ 7 mm. It is reasonable to believe that patients who underwent uterine cavity exploration were less likely to present with RPOC. However, patients with RPOC after uterine cavity exploration will present with a smaller mass than those who did not. One study found that RPOC was higher among patients with third stage placental complications compared to those without these complications (3.7% vs. 0.3%, p < .001) [21].

Another risk factor was previous cesarean section. Patients with an history of one or more cesarean deliveries were more likely to present with sonographic RPOC > 7 mm in the postpartum period (18% vs. 0%, p = .03). Granfors et al., described that RPOC were more prevalent among women with previous cesarean section compared with those with a previous vaginal delivery (3.4% vs. 1.9%; p < .0001) [22]. This finding correlates with the literature and can be explained by previous uterine scarring which exposes a larger area of the uterine cavity that may allow placental tissue to be retained after delivery.

Clinical parameters that correlated with sonographic RPOC > 7 mm included prolonged vaginal bleeding without postpartum hemorrhage (PPH). Studies have shown that RPOC may be a cause of PPH [23]. In this case, the focus is on vaginal bleeding and not PPH. Another important parameter that can help us with the diagnosis is the timing of the evaluation. Patients with a longer interval from delivery to hysteroscopy had smaller sonographic RPOC, usually ≤ 7 mm.

The strengths of this study include that it was conducted in a single center with a standard protocol for follow-up and treatment. In addition, all sonograms were performed by experienced sonographers in a tertiary level hospital. Finally, the waiting period between delivery and hysteroscopy was an average of 78 days. We believe that earlier intervention would find many other RPOC that resolved spontaneously.

The main limitations of the current study are its retrospective nature and relatively small sample size. A larger cohort may have enabled us to demonstrate stronger statistically significant findings.

## Conclusions

Sonographic evaluation after RPOC showed that residua > 7 mm were statistically correlated with positive RPOC in pathology, and PPV of 75% and NPV of 50%. Due to the high NPV and low complication rate of office hysteroscopy, clinicians should consider intervention when any RPOC are measured during sonographic examination to reduce known long-term complications.

## Data Availability

The datasets used and/or analyzed during the current study are available from the corresponding author on reasonable request.

## List of abbreviations

AUC: area under the curve
NPV: negative predictive value
PPH: postpartum hemorrhage
PPV: positive predictive value
ROCs: receiver operating curves
RPOC: retained products of conception
