# Correction: Ultrasound for diagnosis of postpartum retained products of conception—how accurate we are?

**DOI:** 10.1186/s12884-023-05936-4

**Published:** 2023-08-24

**Authors:** Yael Yagur, Liron Jurman, Omer Weitzner, Nissim Arbib, Ofer Markovitch, Zvi Klein, Yair Daykan, Ron Schonman

**Affiliations:** 1 Department of Obstetrics and Gynecology, Meir Medical Center, Kfar Saba, Israel; 2 Faculty of Medicine, Tel Aviv University, Tel Aviv, Israel

**Correction: BMC Pregnancy Childbirth 23, 572 (2023)**

10.1186/s12884-023-05863-4

Following publication of the original article [1], the authors reported an error in affiliations. All authors should be labeled as 1 and 2 and not only 1.

The author group has been updated above and the original article [1] has been corrected.
